# Antigenic Variant of Highly Pathogenic Avian Influenza A(H7N9) Virus, China, 2019

**DOI:** 10.3201/eid2602.191105

**Published:** 2020-02

**Authors:** Wenming Jiang, Guangyu Hou, Jinping Li, Cheng Peng, Suchun Wang, Shuo Liu, Qingye Zhuang, Liping Yuan, Xiaohui Yu, Yang Li, Jingjing Wang, Hualei Liu

**Affiliations:** China Animal Health and Epidemiology Center, Qingdao, China

**Keywords:** Influenza virus, H7N9, antigenicity, variant, influenza, viruses, highly pathogenic avian influenza, HPAI, China

## Abstract

In China, influenza A(H7N9) virus appeared in 2013, then mutated into a highly pathogenic virus, causing outbreaks among poultry and cases in humans. Since September 2017, extensive use of the corresponding vaccine, H7-Re1, successfully reduced virus prevalence. However, in 2019, a novel antigenic variant emerged, posing considerable economic and public health threats.

Since mid-2016, influenza A(H7N9), a highly pathogenic avian influenza (HPAI) virus, has led to ≈17 outbreaks in poultry in China (*1**–**3*). Extensive use of the corresponding vaccine, H7-Re1, substantially reduced the prevalence of H7N9 viruses (*4*,*5*). However, in early 2019, active surveillance detected the unprecedented and rapid emergence of a novel HPAI H7N9 virus antigenic variant in several regions of China.

Since 2013, a total of 1,567 cases of human infection with novel H7N9 viruses, associated with a high mortality rate, have been reported in China (*6*). Studies on circulating H7N9 viruses have suggested that they originated from poultry (*7*). However, strains isolated from birds at live bird markets displayed low pathogenicity in poultry (*8*). In early 2017, several outbreaks caused by HPAI H7N9 viruses in poultry were reported. To control infection of poultry and reduce the risk for human exposure to H7N9 virus, development and national use of an inactivated vaccine, H7-Re1, with hemagglutinin (HA) and neuraminidase (NA) genes derived from A/pigeon/Shanghai/S1069/2013 (H7N9), has since September 2017 substantially decreased prevalence of H7N9 viruses among poultry and humans (*5*). In December 2018, on the basis of surveillance findings, the original vaccine was replaced with the H7-Re2 vaccine, with HA and NA genes derived from A/chicken/Guangdong/SD098/2017(H7N9).

In 2019, during active surveillance for avian influenza infection in China, we identified 7 strains of H7N9 viruses from 4,226 chicken swab samples. We isolated the strains by inoculating them into 10-day-old specific-pathogen–free chicken embryos and confirmed their identification via reverse-transcription PCR and sequencing. Viruses were from Hebei and Liaoning Provinces and designated A/chicken/China/FQ2/2019(H7N9) (FQ2), A/chicken/China/QHD1/2019(H7N9) (QHD1), A/chicken/China/DL614/2019(H7N9) (DL614), A/chicken/China/AS1/2019(H7N9) (AS1), A/chicken/China/WYG1/2019(H7N9) (WYG1), A/chicken/China/HD1/2019(H7N9) (HD1), and A/chicken/China/DL1/2019(H7N9) (DL1). After determining the HA and NA sequences of the viruses, we deposited the data in GenBank (accession nos. MN700030–43).

According to the deduced amino acid sequence of HA, all strains contained multiple basic amino acids (PKRKRTAR/GLF) at the cleavage site, suggestive of high pathogenicity. This theory was further confirmed by analysis of the intravenous pathogenicity index. In chickens, pathogenicity of the strains was high (index values 2.18, 2.32, 2.28, 2.18, 2.26, 2.30, and 2.36), but in ducks, pathogenicity was low. Although the viruses had replicated in the internal organs (brain, lungs, spleen, liver, intestine, and kidneys) of inoculated ducks on postinoculation days 3 and 5, no deaths or signs of infection were observed within 14 days after inoculation (Appendix Table).

We determined that the amino acid residues at the receptor-binding site of HA proteins are A138, V186, P221, and Q226 (H3 numbering), which suggests that these viruses could bind receptors in birds and humans (*9*). The phylogenetic tree based on the HA gene showed that all strains belong to the highly pathogenic H7N9 clade but are clearly distinguishable from HPAI H7N9 viruses isolated in 2017 and 2018 (Appendix Figure).

Amino acid identities of the HA gene segments of these strains were 95.8%–96.5% identical to those of H7-Re1 (92.7%–93.7% for HA1) and 97.4%–98.0% identical to those of H7-Re2 (96.2%–97.2% for HA1). To evaluate the antigenicity and protective efficacy of the H7-Re2 vaccine, we vaccinated specific-pathogen–free chickens with H7-Re2 and rFQ2 (a reverse genetic recombinant carrying HA and NA of FQ2 with internal gene segments of PR8). FQ2 and DL1 viruses were selected for subsequent experiments. Cross-reactive hemagglutination inhibition titers of H7-Re2 antiserum against FQ2 and DL1 viruses were 4.5–4.6 log_2_ lower than those against the homologous H7-Re2 antigen. In contrast, cross-reactive HI titers of antiserum against H7-Re2 antigens from rFQ2 virus did not differ markedly from those against the 2 homologous H7N9 viruses. These results indicate that the FQ2 and DL1 viruses exhibited rapid antigenic drift and distinct antigenicity relative to the H7-Re2 vaccine strain.

During the 10-day observation period after challenge, H7-Re2–vaccinated birds displayed clinical signs of infection, such as depression, huddling, and decreased consumption of feed and water. Moreover, shed virus was detected in tracheal and cloacal swab samples from all experimentally inoculated chickens on postchallenge days 3 and 5. Only 40.0% of the challenged chickens survived, indicating that the H7-Re2 vaccine had a poor protective effect against FQ2 and DL1.

All rFQ2-vaccinated birds survived with no clinical signs of infection. In addition, no virus shedding was detected in tracheal or cloacal swab samples from any rFQ2-vaccinated chickens on postchallenge days 3 and 5 (Table). Of note, antiserum against the rFQ2 virus showed a broader spectrum of reactivity to other viruses, including H7-Re2, indicating that recombinant rFQ2 offers a better alternative for vaccine development.

**Table:** Efficacy of H7-Re2 vaccine against highly pathogenic avian influenza A(H7N9) viruses in chickens, China, 2019*

Vaccine	Challenge virus	Mean HI titer 21 d after vaccination (log_2_)			Virus shedding					Survival rate
					Postchallenge day 3			Postchallenge day 5		
		Challenge virus	H7-Re2		Trachea	Cloaca		Trachea	Cloaca	
H7-Re2	FQ2	3.2 ± 0.6	7.7 ± 0.5		8/8 (2.6 ± 0.4)	8/8 (2.4 ± 0.3)		4/4 (2.3 ± 0.3)	4/4 (2.5 ± 0.4)	4/10
	DL1	3.3 ± 0.5	7.9 ± 0.4		8/8 (2.8 ± 0.3)	8/8 (2.5 ± 0.3)		4/4 (2.2 ± 0.4)	4/4 (2.3 ± 0.3)	4/10
rFQ2	FQ2	7.6 ± 0.6	7.0 ± 0.5		0/10	0/10		0/10	0/10	10/10
	DL1	7.3 ± 0.5	6.8 ± 0.4		0/10	0/10		0/10	0/10	10/10
Control	FQ2	<1	<1		4/4 (4.8 ± 0.5)	4/4 (4.5 ± 0.4)		NA	NA	0/10
	DL1	<1	<1		4/4 (4.7 ± 0.4)	4/4 (4.9 ± 0.5)		NA	NA	0/10

*HI, hemagglutination inhibition; NA, not applicable because of death of chickens.

In China, vaccination plays a decisive role in the prevention and control of H7N9 virus–mediated infection. Earlier mass vaccination of poultry with H7-Re1 successfully induced a sharp decline in H7N9 infection prevalence among poultry and humans. However, as of 2019, H7N9 variants have surfaced, posing a considerable economic and public health threat and highlighting the urgent need for new antigen-matched vaccines and more productive measures to eliminate highly pathogenic H7N9 viruses.

Appendix Supplemental data from study of antigenic variant of highly pathogenic avian influenza A(H7N9) virus in China, 2019.
