# Supplementary material for: Antigenic Variant of Highly Pathogenic Avian Influenza A(H7N9) Virus, China, 2019
Source: Emerg Infect Dis. 2020 Feb;26(2):379–80. doi: 10.3201/eid2602.191105 (PMC6986829; doi:10.3201/eid2602.191105)
Supplement: Appendix — Supplemental data from study of antigenic variant of highly pathogenic avian influenza A(H7N9) virus in China, 2019. [file 19-1105-Techapp-s1.pdf]

Article DOI: https://doi.org/10.3201/eid2602.191105

Antigenic Variant of Highly Pathogenic Avian Influenza A(H7N9) Virus, China, 2019

Appendix

Appendix Table. Virulence and shedding of H7N9 highly pathogenic avian influenza viruses in ducks

			Virus isolation						Virus shedding		
			Positive number/total number (Mean Ct)						Number shedding/total number (Mean Ct)		
Isolate	Day post challenge	No. necropsy	Brain	Liver	Spleen	Lung	Intestine	Kidney	Tracheal	Cloacal	Seroconversion (positive/total)
FQ2	3	5	1/5	2/5	2/5	2/5	5/5	2/5	10/10	10/10	10/10
	5	5	1/5	2/5	2/5	2/5	5/5	2/5	10/10	10/10	
DL1	3	5	1/5	2/5	3/5	3/5	5/5	2/5	10/10	10/10	10/10
	5	5	1/5	2/5	3/5	3/5	5/5	2/5	10/10	10/10	

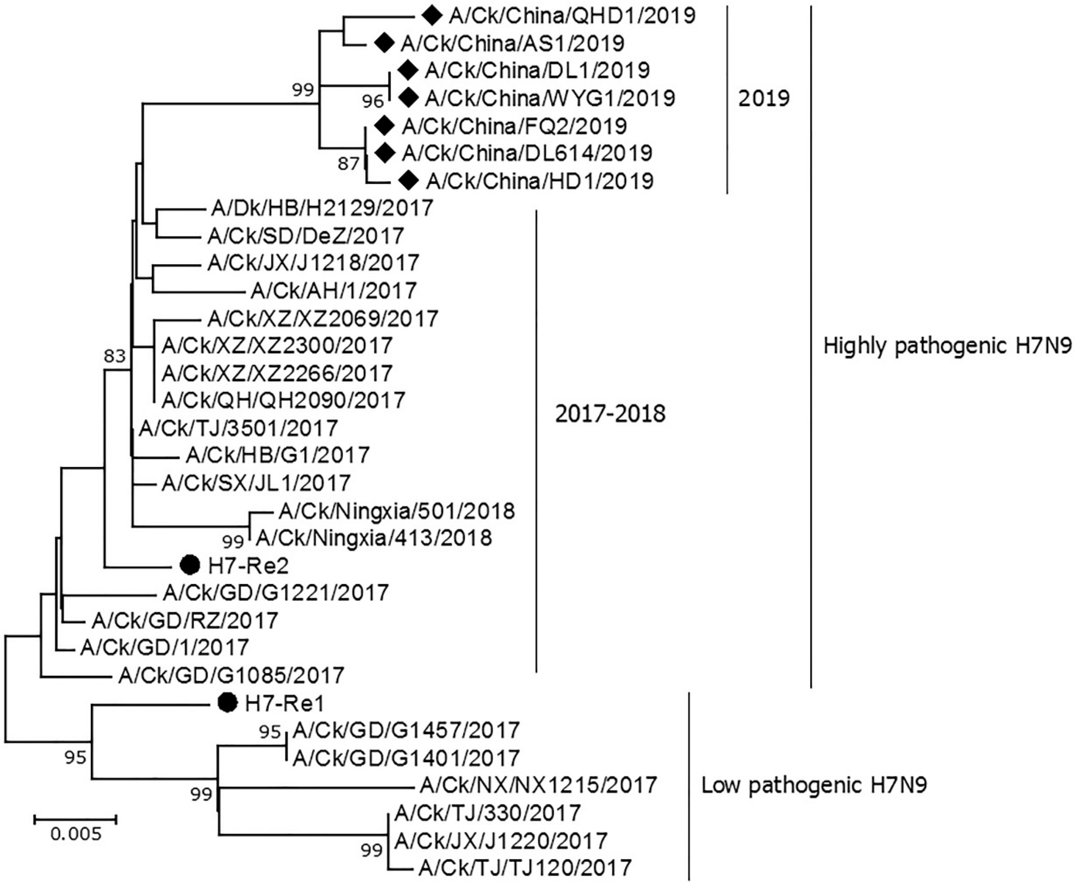

Appendix Figure. Phylogenetic trees of the hemagglutinin gene of H7N9 avian influenza viruses. Trees were constructed with MEGA6.05 software using the neighbor-joining method. Bootstrap analysis was performed with 1,000 replications. "◆"represents viruses isolated in 2019 and "●"represents vaccine strains. Scale bars indicate the number of nucleotide substitutions per site.
